# Prior year hospital admission predicts 30-day hospital readmission after spine surgery

**DOI:** 10.1016/j.xnsj.2025.100750

**Published:** 2025-06-10

**Authors:** Ryan T. Lin, Rahul Ramanathan, Jonathan F. Dalton, Christopher J. Como, Isaac Lee, Christopher Gonzalez, Melissa Yunting Tang, Anthony A. Oyekan, Audrey Y. Chang, Michael Spitnale, Jeremy D. Shaw, Joon Y. Lee, Richard A. Wawrose

**Affiliations:** a Department of Orthopaedic Surgery, University of Pittsburgh, Pittsburgh PA, United States; b Pittsburgh Orthopaedic Spine Research (POSR) Group, Pittsburgh PA, United States; c The Orland Bethel Family Musculoskeletal Research Center (BMRC), Pittsburgh PA, United States; d Intermountain Health, Salt Lake City UT, United States

**Keywords:** Machine learning, Hospital readmission, Spine surgery, Risk stratification, Postoperative complications, Quality improvement

## Abstract

**Background:**

Reducing hospital readmissions postspine surgery is crucial for improving care quality. Currently, no specific scoring system exists to stratify patients by readmission risk in spine surgery. The objective of this study was to evaluate risk factors for 30-day hospital readmission following elective decompressive spine surgery and develop an ML-based risk-scoring system.

**Methods:**

653 patients undergoing elective decompressive spine surgery (with or without fusion) at a single academic center were retrospectively reviewed. Data collected included demographics, surgical details, preoperative admissions, and complications. Univariate logistic and Cox regression analyses identified significant predictors. A supervised ML-based risk scoring system was developed and compared against the institution’s standard risk calculator.

**Results:**

Of 653 patients, 44 (6.7%) experienced a 30-day readmission. Predictors of readmission included prior-year hospital admission, age-adjusted Charlson Comorbidity Index (CCI), traumatic or thoracic surgery, postoperative complications, and length of stay. An ML-based scoring system stratified patients into Low-risk (0–4 points; 2.16% readmission), Moderate-risk (5–9 points; 7.25%), High-risk (10–14 points; 21.57%), and Extreme-risk (15+ points; 39.47%) groups. Compared to the standard hospital score, the new model demonstrated superior performance: higher C-index (0.79 vs. 0.71), higher Somers’ D (0.56 vs. 0.41), and lower Akaike Information Criterion (AIC) (516.78 vs. 546.65).

**Conclusions:**

Preoperative admission within 1 year prior to elective decompressive spine surgery significantly predicts 30-day readmission risk. Additional factors include age-adjusted CCI, traumatic or thoracic surgery, complications, and length of hospital stay. The proposed ML-based scoring system demonstrated superior predictive performance compared to the standard hospital readmission risk calculator, serving as a potentially valuable internal quality improvement tool pending external validation.

## Introduction

Approximately 900,000 patients undergo spine surgery on an annual basis in the United States alone, with the number continuing to rise due to the aging population [1,2]. Although many commonly performed spine surgery techniques are associated with an excellent safety profile, complications can arise at higher frequencies for more complex surgical indications such as infection, trauma, or revision [[3], [4], [5]]. These complications may lead to unplanned emergency department visits or hospital readmissions—both of which contribute significantly to an estimated healthcare burden of $17.6 billion annually [6,7]. The Centers for Medicare and Medicaid Services (CMS) have implemented penalties for hospitals with higher-than-expected readmission rates, as it is believed that many of these readmissions are preventable and reflect suboptimal care [8].

There has been a growing focus on identifying factors that may predict and mitigate the risk of readmission following spine surgery. Previous literature has found that 30-day unplanned hospital readmission occurs at a rate of about 5.5% to 9.4% in the postoperative setting of spine surgery [[9], [10], [11]]. Overall, the most common reasons for readmission are procedure-related, including concerns for infection and pain, but medical concerns such as drug reaction or overdose were also significant contributors [9,10]. A systemic review of 30-day readmissions after spine surgery and found numerous factors that contribute to readmission risk, including surgery type (2.5% readmission rate for anterior cervical discectomy and fusion) and indication category (14.2% readmission rate for primary and metastatic spine tumors) [[11], [12], [13]]. Prior hospital admission is a known risk factor for readmission in the trauma setting, but its impact on readmission rates after spinal decompressive surgery is not well understood [[14], [15], [16]]. Additionally, although there has been significant research to identify risk factors for 30-day unplanned readmission after spine surgery, strategies to predict and decrease readmission in patients undergoing spine surgery are lacking [[17], [18], [19], [20]].

The purpose of this study was to investigate risk factors for 30-day readmission after spine surgery, particularly the impact of preoperative hospital admission. A secondary goal was to create a novel scoring system and risk calculator to stratify patients by 30-day readmission risk using machine learning-based modeling. It was hypothesized that patients with hospital admission for any reason within a year of their index spine surgery would be more likely to have an unplanned hospital readmission within 30 days postoperatively.

## Methods

This Institutional Review Board-approved retrospective cohort study included adult patients (≥18 years) who underwent primary or revision spine surgery for traumatic or degenerative indications (with or without fusion) by 3 fellowship-trained orthopaedic spine surgeons at a single academic institution from June 24, 2020, to June 28, 2021. Eligible patients had at least 1 year of preoperative hospital admission data available. Patients were excluded for missing postoperative follow-up data or procedures unrelated to spine decompression or fusion.

Patients were stratified into 2 groups based on the occurrence of an unplanned hospital readmission within 30 days of their index spine surgery. The 30-day readmission group included patients who experienced at least 1 unplanned all-cause hospital admission within 30 days postoperatively. The nonreadmission group consisted of patients who did not experience any unplanned admission during that same postoperative period. Planned admissions (e.g., for steroid injections, wound checks, physical therapy) were excluded.

Demographic and preoperative data collected from electronic medical records (EMR) included age, gender, body mass index (BMI), Charlson Comorbidity Index (CCI), and prior year hospitalizations. Age-adjusted CCI was calculated by adding 1 point per decade after age 40 [21]. Surgical and postoperative variables included type of surgery (decompression alone, fusion, revision), surgical indication (degenerative disease, trauma, infection), surgical approach, interbody device use, estimated blood loss (EBL), hospital length of stay (LOS), and postoperative complications, including those requiring revision surgery. The primary outcome was unplanned all-cause 30-day hospital readmission after the index procedure; planned admissions (e.g., hardware removal, steroid injections, palliative care, physical therapy) were excluded. Secondary outcomes included descriptive statistics on the etiology of 30-day readmissions.

### Covariate selection

Covariates analyzed included sex, age, BMI, age-adjusted Charlson Comorbidity Index (CCI), surgical indication, surgical approach, interbody device usage, spine level, surgery type (decompression, fusion, revision), prior year hospital admissions, estimated blood loss (EBL), primary versus revision procedure, postoperative hospital complications, complications requiring additional surgery, and hospital length of stay (LOS). Univariate logistic regression analyses identified covariates with significance (p < .05) for inclusion in the final Cox regression models. Models were adjusted for sex, surgical indication, and surgical level unless the covariate of interest was included in the adjustment variable. If a covariate lost significance after time-to-event analysis, confounding was defined as a change in odds ratio greater than 20% compared to the previous model.

### Statistical methods and machine learning approach

Univariate analysis utilized Student’s t-test for continuous variables and Chi-squared or Fisher’s exact test for categorical variables. Survival analysis was performed using Cox proportional hazards models. The time-to-event variable as number of days to readmission if there was readmission or were censored at time of last follow-up if there was no readmission event. The proportional hazards assumption was tested using Schoenfeld residuals, and no violations of the proportional hazards assumption were identified during analysis. Final Cox-regression models were validated using log-likelihood ratio tests and goodness-of-fit was tested via Cox-Snell residuals. Variable inflation factor (VIF) was also calculated for each analysis to evaluate for multicollinearity and only variables with VIF less than 5 were included. All statistical analyses were performed using Stata version 18.0 (StataCorp LLC, College Station, TX, USA) and a p-value < .05 was considered significant.

### 30-day readmission risk score for decompression- and fusion-related spine surgery and comparison to standardized hospital readmission risk score—testing of ML models

Continuous predictors (age-adjusted CCI, prior hospital readmissions, and LOS) were categorized based on predefined criteria and analyzed using univariate Cox regression. After validation with Cox-Snell residuals, hazard ratios from final models determined the readmission risk score. Bivariate logistic regression then evaluated the association between the calculated risk scores and risk categories with actual 30-day hospital readmissions. From these models, 2 receiver operating curves (ROC) were created and the areas under the receiver operator curves (AUC) were reported. AUC calculations were performed using the same dataset employed for model development, without external validation at time of publication.

The study’s risk score was compared to the institution’s standardized hospital readmission risk score, developed with the XGBoost machine learning library, predicting 7- and 30-day readmissions. This comparator model was trained on 1.1 million discharges across 17 hospitals using prior hospital utilization, demographic variables, chief complaints, comorbidities, medication use, laboratory values, and census-derived social determinants of health. The model categorizes readmission risk into subgroups (lowest, lower, medium, higher, highest risk), triggering systemwide discharge follow-up interventions. It was prospectively validated within the author’s healthcare system and shown to accurately predict readmission risk [22].

Comparison metrics used between the 2 models included concordance index (C-index), Somers’ D, and Akaike Information Criterion (AIC). C-index values <0.6 indicate poor discrimination, 0.6 to 0.75 moderate, and >0.75 strong predictive discrimination [23]. Somers’ D statistic assesses predictive accuracy of model covariates for readmission risk, ranging from −1 (inverse prediction) to 1 (perfect prediction), with values farther from 0 indicating stronger prediction. Finally, AIC is a relative measure for model fit, with lower AIC values indicating model fit between model complexity and goodness-of-fit.

## Results

**Fig. 1:**
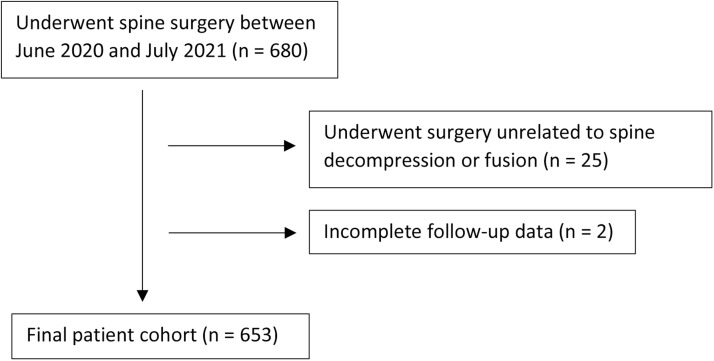
Patient selection flowchart.

A total of 680 patients met inclusion criteria; 25 (3.7%) were excluded for surgeries unrelated to fusion or decompression, and 2 (0.3%) for incomplete follow-up (Fig. 1). The final cohort consisted of 653 patients, with 44 (6.7%) experiencing an unplanned 30-day readmission. Demographic variables (sex, age, BMI, and CCI) and surgical characteristics (surgery type, primary vs. revision, intraoperative blood loss) showed no significant differences between groups. Compared to patients without readmission, those who were readmitted within 30 days had significantly higher rates of prior year hospital admissions (0.6 ± 1.0 vs. 0.2 ± 0.7, p = .003), higher age-adjusted CCI (3.9 ± 1.7 vs. 3.2 ± 1.8, p = .046), and a greater incidence of traumatic indications (31.8% vs. 14.6%, p = .002). Readmitted patients also had longer hospital stays (LOS, 8.7 ± 7.6 vs. 4.5 ± 5.1 days, p < .0001), more postoperative in-hospital complications (65.9% vs. 27.6%, p < .0001), and more frequent postoperative in-hospital complications requiring reoperation (22.7% vs. 2.6%, p < .0001) (Table 1). In contrast, patients in the 30-day readmission group, when compared to the nonreadmission group, had fewer surgeries with a degenerative indication (59.1% vs. 83.1%, p < .0001) and lower rate of discectomy procedures (2.3% vs. 12.5%, p = .043). Surgical approach, multiple indications, and interbody device use showed no significant associations with readmission (all p > .05) (Table 1).

**Table 1:** Comparison of demographic, surgical, and postoperative hospital characteristics between thirty-day hospital readmission versus control group.

	Readmission group (*n* = 44)	Control group (*n* = 609)	p-value
Sex, n (%)			
Male	27 (61.4)	328 (53.9)	.334
Female	17 (38.6)	281 (46.1)	
Age, mean ± SD	63.0 ± 12.3	59.1 ± 14.0	.085
BMI, mean ± SD	29.3 ± 4.8	30.4 ± 6.1	.233
CCI, mean ± SD	1.0 ± 1.4	0.8 ± 1.2	.260
Age-adjusted CCI, mean ± SD	3.9 ± 1.7	3.2 ± 1.8	**.046**
Revision procedure, n (%)	10 (22.7)	165 (27.1)	.528
Surgery type			.698
Laminectomy, n (%)	38 (86.4)	458 (75.2)	.094
PSF, n (%)	32 (72.7)	365 (59.9)	.093
Cervical ASF, n (%)	0 (0)	10 (1.6)	.392
ALIF, n (%)	3 (6.8)	42 (6.9)	.984
ROH, n (%)	7 (15.9)	67 (11.0)	.321
I&D, n (%)	2 (4.6)	9 (1.5)	.127
ACDF, n (%)	3 (6.8)	80 (13.1)	.224
Diskectomy, n (%)	1 (2.3)	76 (12.5)	**.043**
XLIF, n (%)	2 (4.6)	12 (2.0)	.255
Corpectomy, n (%)	2 (4.6)	25 (4.1)	.887
PLIF, n (%)	1 (2.3)	7 (1.2)	.513
Multiple surgical approaches, n (%)	4 (9.1)	55 (9.0)	1.00
Use of interbody, n (%)	9 (20.5)	141 (23.2)	.681
Surgery indications			**.027**
Degenerative, n (%)	26 (59.1)	506 (83.1)	**<.0001**
Trauma, n (%)	14 (31.8)	89 (14.6)	**.002**
Infection, n (%)	3 (6.8)	13 (2.13)	.086
Tumor, n (%)	0 (0)	5 (0.8)	.546
Deformity, n (%)	4 (9.1)	20 (3.3)	.071
Hardware failure, n (%)	0 (0)	8 (1.3)	.444
Multiple surgical indications, n (%)	3 (6.8)	30 (4.9)	.481
Surgery level			.080
Cervical, n (%)	13 (29.6)	184 (30.2)	.926
Thoracic, n (%)	17 (38.6)	129 (21.2)	**.007**
Lumbar, n (%)	29 (65.9)	412 (67.7)	.812
Sacral, n (%)	9 (20.5)	118 (19.4)	.861
Number of prior year hospital visits, mean ± SD	0.6 ± 1.0	0.2 ± 0.7	**.003**
Postoperative in-hospital complication, n (%)	29 (65.9)	168 (27.6)	**<.0001**
Postoperative in-hospital complication requiring surgery, n (%)	10 (22.7)	16 (2.6)	**<.0001**
Estimated blood loss during procedure, mean ± SD	481.5 ± 440.0	377.4 ± 550.5	.221
Length of stay (days), mean ± SD	8.7 ± 7.6	4.5 ± 5.1	**<.0001**

Significant p-values are bolded.

Multiple surgical approaches were defined as requiring access to the spine from different anatomical directions (i.e., anterior, posterior, lateral). Interbody fusion surgery was defined as those undergoing ALIF, ACDF, XLIF, or PLIF.

Univariate logistic regression analysis of covariates revealed predictors of 30-day hospital readmission, including age-adjusted CCI (p = .047), degenerative surgical indication (p < .0001), trauma surgical indication (p = .003), thoracic surgery level (p = .009), number of prior year hospital visits (p = .006), postoperative in-hospital complication (p < .0001), postoperative in-hospital complication requiring surgery (p < .0001), and LOS (p = .001). Of the covariates identified on univariate analysis, only discectomy surgery did not remain statistically significant (p = .075) (Table 2).

**Table 2:** Univariate logistic regression analysis of covariates as predictors of 30-day hospital readmission.

	Odds Ratio	95% CI	p-value
Age-adjusted CCI	1.19	1.00–1.41	**.047**
Surgery—Diskectomy	0.16	0.02–1.20	.075
Surgical indication—Degenerative	0.29	0.16–0.56	**<.0001**
Surgical indication—Trauma	2.73	1.39–5.34	**.003**
Location—Thoracic	2.34	1.24–4.43	**.009**
Number of prior year hospital visits	1.51	1.13–2.02	**.006**
Postoperative in-hospital complication	5.08	2.65–9.70	**<.0001**
Postoperative in-hospital complication requiring surgery	10.90	4.60–25.82	**<.0001**
Length of stay (days)	1.07	1.03–1.11	**.001**

Significant p-values are bolded.

Final univariate survival analyses using categorical prognostic risk factors, adjusted for sex, surgical indication, and level, showed significant predictors of 30-day readmission: prior hospital admission within 1 year (HR: 1.51, 95% CI: 1.09–2.09, p = .014), higher age-adjusted CCI (HR: 1.2, 95% CI: 1.0–1.4), trauma indication (OR: 2.46, 95% CI: 1.3–4.6), infection indication (OR: 2.6, 95% CI: 1.0–6.6), thoracic surgery location (OR: 2.4, 95% CI: 1.3–4.4), postoperative complication before discharge (OR: 5.1, 95% CI: 2.8–9.5), postoperative complication requiring additional surgery (OR: 7.1, 95% CI: 3.5–14.2), and increased LOS (OR: 1.1, 95% CI: 1.03–1.07) (Table 3).

**Table 3:** Categorical predictors of 30-day hospital readmissions after spine surgery with univariate Cox regression analysis.

	Hazard ratio	95% CI	p-value
Age-adjusted CCI groups (0, 1–2, 3–4, 5–6, 7+)	1.44	1.02–2.03	**.036**
Surgical indication			
Degenerative	0.31	0.16–0.60	**<.0001**
Trauma	2.55	1.30–5.00	**.006**
Location—Thoracic	1.97	1.04–3.77	**.039**
Number of prior year hospital visits groups (0, 1, 2, 3+)	1.51	1.09–2.09	**.014**
Postoperative in-hospital complication	4.55	2.43–8.53	**<.0001**
Postoperative in-hospital complication requiring surgery	7.47	3.62–15.43	**<.0001**
Length of stay groups (0–2, 3–5, 6–10, 11+ days)	2.57	1.78–3.70	**<.0001**

Age-adjusted CCI and LOS were categorized in ordinal fashion with higher scores and in-hospital LOS having elevated risk. All variables are adjusted for sex, surgical indication, and surgical level, if applicable.

Significant p-values are bolded.

### Derivation of readmission risk score calculator and comparison to standardized hospital readmission risk score

**Fig. 2:**
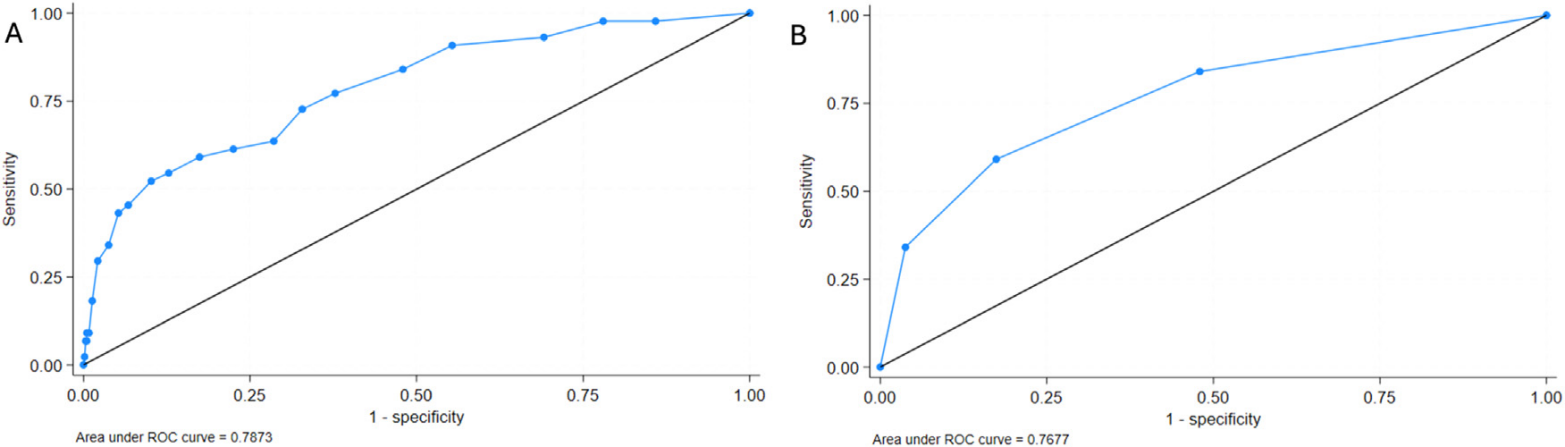
Receiver operating curves derived from logistic regression Model A based on risk score values (A) and regression Model B based on risk score groups (B) to predict thirty-day readmission after spine surgery.

**Fig. 3:**
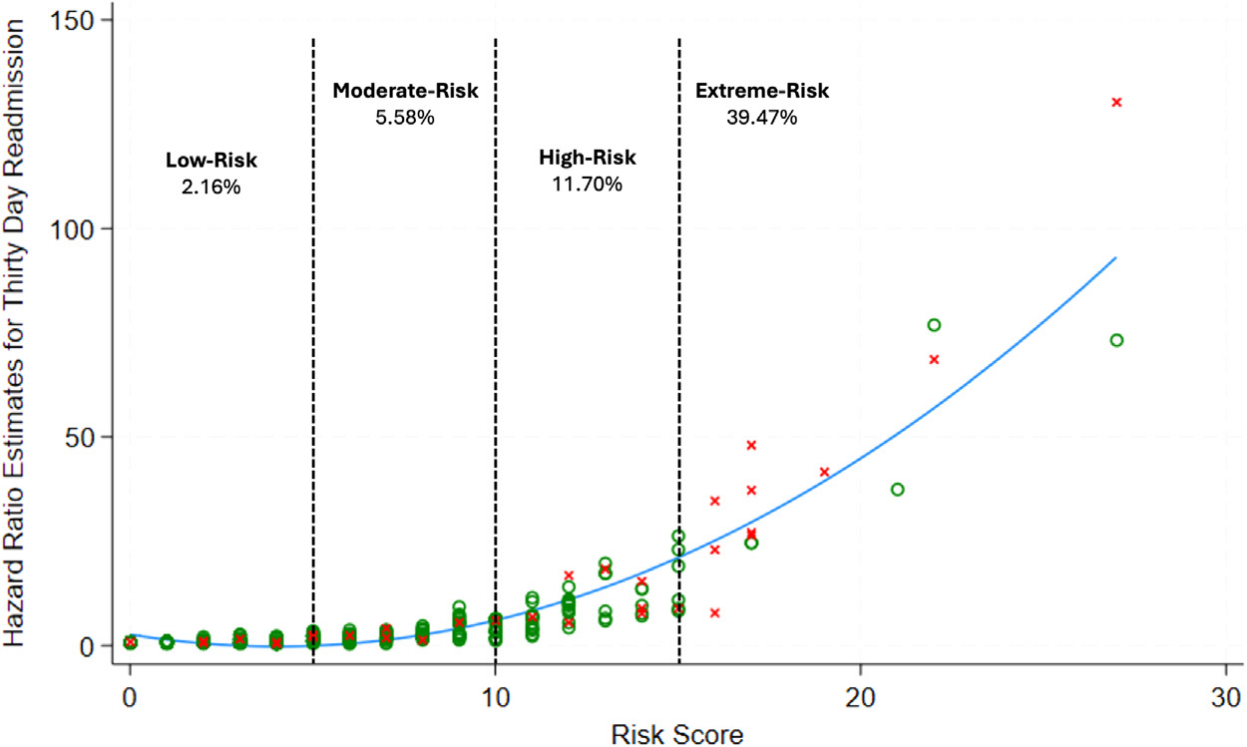
Illustrates how this study’s proposed thirty-day readmission risk score correlates with predicted hazards estimated from final univariate cox regression models. Red “x” denotes patients who experienced thirty-day hospital readmission. Green “o” patients who did not experience 30-day hospital readmission. Each risk group ranging from low- to extreme-risk ask displays the proportion of individuals who experienced thirty-day hospital readmission.

Final risk score categories and scoring criteria ranged from 0 to 30 points (Table 4). Bivariate logistic regression analysis revealed that risk for 30-day readmission increased by 24% with every 1-unit increase in risk score (OR: 1.24, 95% CI: 1.16–1.31, p < .0001) (Table 5). The model based on risk score was shown to be predictive (AUC = 0.79) of 30-day readmissions based on ROC results (Fig. 2A). There was a strong association observed between risk scores and 30-day readmission risk from postestimation analysis (Fig. 3). Risk categories were stratified into risk subgroups: Low-Risk (0–4), Moderate-Risk (5–9), High-Risk (10–14), and Extreme-Risk (15 and greater). In these subgroups, 2.16% of Low-Risk, 5.58% of Moderate-Risk, 11.70% of High-Risk, and 39.47% of Extreme-Risk patients experienced 30-day hospital readmission after spine surgery (Fig. 3). Risk score categories were also shown to be predictive of 30-day readmission (AUC = 0.77) according to the ROC (Fig. 2B).

**Table 4:** Risk score based on preoperative and in-hospital predictors of 30-day readmission.

Prognostic factors	Points
Age-adjusted CCI	
0	0
1–2	1
3–4	2
5–6	3
7+	4
Surgical indication	
Degenerative	−1
Trauma	3
Surgical location—Thoracic	2
Number of prior year hospital visits	
0	0
1	1
2	2
3+	3
Postoperative in-hospital complication	5
Postoperative in-hospital complication requiring surgery	7
In-hospital length of stay (days)	
0–2 days	0
3–5 days	2
6–10 days	4
11+ days	6

**Table 5:** Risk score groups as predictors of 30-day readmissions.

*Logistic regression Model A*			
Risk score values	Odds ratio	95% CI	p-value
	1.24	1.16–1.31	**<.0001**
*Logistic regression Model B*			

Risk score groups	Odds ratio	95% CI	p-value

*Low-risk*	1	*(Reference)*	*(Reference)*
*Moderate-risk*	2.67	1.02–7.03	**.045**
*High-risk*	6.00	2.26–15.96	**<.0001**
*Extreme-risk*	29.53	10.95–76.64	**<.0001**

Model A demonstrates univariate logistic regression outcomes when analyzing risk score values as a continuous variable predictor for 30-day readmissions. Model B analyzes univariate analysis of risk score groups as a categorical variable predictor of thirty-day readmissions.

Significant p-values are bolded.

**Fig. 4:**
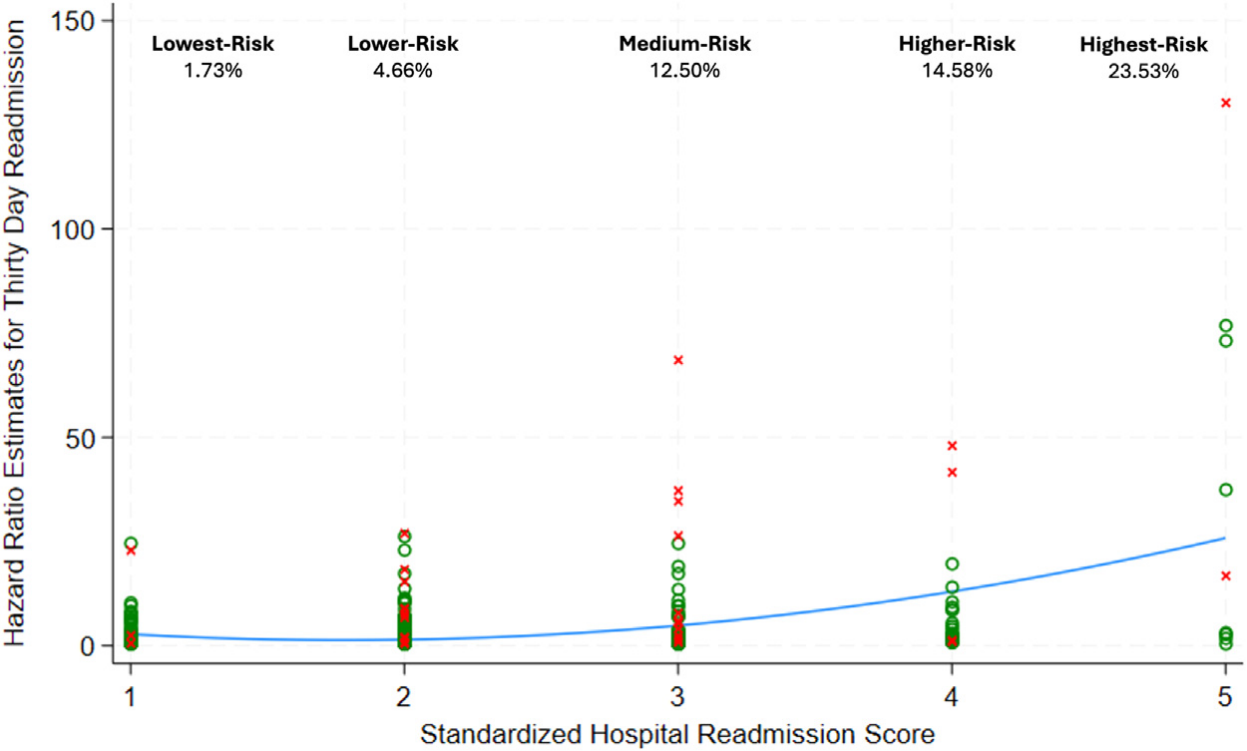
The standardized hospital readmission score for thirty-day readmissions with predicted hazards estimated from final univariate cox regression models.

This study’s proposed risk score had a higher C-index value (0.79 vs. 0.71), higher Somers’ D (0.56 vs. 0.41), and lower AIC (516.78 vs. 546.65) compared to the institutional Standardized Hospital Readmission Score (Table 6). The standardized hospital readmission risk score categories had 1.73% of lowest-risk, 4.66% of lower-risk, 12.50% of medium-risk, 14.58% of higher-risk, and 23.53% of highest-risk patients experiencing 30-day hospital readmission after spine surgery (Fig. 4). Fig. 4 also displays the association between Standardized Hospital Readmission Score categories and 30-day readmission risk from postestimation analysis.

**Table 6:** Comparison between this study’s proposed risk score and the author’s institutional standardized hospital readmission score for predicting 30-day readmission after spine surgery.

	C-index (95% CI)	Somers' D	AIC
Risk score	0.79 (0.71–0.86)	0.56	516.78
Standardized hospital readmission score	0.71 (0.64–0.78)	0.41	546.65

### Etiology of 30-day hospital readmissions

The etiology of 30-day hospital readmissions is displayed in Table 7. There were 21/44 readmissions classified as surgical (47.73%) and 23/44 readmissions classified as medical (52.27%). The most common surgical reasons for hospital readmissions within 30 days after spine surgery included new back pain or weakness (*n* = 7, 15.91%) and surgical site infection or wound dehiscence (*n* = 6, 13.64%). The most common medical reasons included deconditioning (*n* = 5, 11.36%) and nonsurgical site infection (*n* = 3, 6.82%).

**Table 7:** Etiology of 30-day hospital readmissions.

	Readmission (*n* = 44)	
	N	Percent
Surgical	21	47.73%
Abscess	1	2.27%
Dural tear/CSF leak	2	4.55%
Epidural hematoma	2	4.55%
Implant migration	2	4.55%
New back pain/weakness	7	15.91%
Persistent stenosis	1	2.27%
Surgical site infection/wound dehiscence	6	13.64%
Medical	23	52.27%
Cancer	2	4.55%
Cardiac event	2	4.55%
Constipation	1	2.27%
DVT	1	2.27%
Deconditioning	5	11.36%
Drug-related complication	1	2.27%
GI bleed	2	4.55%
Neuro	2	4.55%
Nonsurgical site infection	3	6.82%
Respiratory failure	2	4.55%
Sepsis	1	2.27%
UTI	1	2.27%

## Discussion

The results support the hypothesis that hospital admission within a year prior to decompressive spine surgery (with or without fusion) significantly increases risk of postoperative readmission. Other predictive risk factors include age-adjusted CCI, trauma indication, thoracic spine surgery, postoperative complications, complications requiring surgery, and hospital LOS. These factors were utilized to create a risk assessment score to identify high-risk spine surgery patients, guiding targeted postoperative interventions to potentially reduce readmission and healthcare burden [[24], [25], [26], [27]]. The author’s proposed risk score was also shown to be superior to their institution’s standardized hospital readmission risk score based on discriminative power, predictive accuracy, and model fit based on this study’s population.

Importantly, this spine-specific model is tailored to the institution’s patient population to support local quality improvement and care optimization.

Prior literature has also identified preoperative hospitalization as a risk factor for unplanned postoperative readmission following both orthopedic and general surgeries. Indeed, orthopedic literature has shown that being hospitalized within the year prior to surgical fixation of either a hip or femur fracture was associated with 7.2 times higher odds of postfixation 90-day readmission [16]. The present study affirms the validity of these findings as well as their generalizability to spine surgery. Similarly, in the field of cardiac surgery, 1 group found that prior hospitalization was independently associated with postoperative infections, which is a known risk factor for postoperative unplanned hospital readmissions [28,29]. It has also been shown the duration of preoperative hospital length of stay was also an independent predictor of postoperative infections and unplanned postoperative hospital readmission [30]. Although the current study did not examine the impact of length of preoperative hospitalization, future research should evaluate this variable in the setting of readmission risk after spine surgery.

Although preoperative hospitalizations have been studied as a risk factor for readmission in other surgical fields, their significance in spine surgery remains underexplored. Readmission risk is critical in spine surgery, given that complications can cause neurologic deficits and substantial morbidity or mortality [31,32]. Rohrer et al. found spine surgery itself independently increased readmission odds nearly 7-fold compared to other orthopedic procedures, suggesting generalized risk calculators may underappreciate the unique risks of spine surgery [33]. Previous studies have highlighted key risk factors in spine surgery, such as increased hospital length of stay (LOS) and surgical complications [34]. Lange et al. similarly found prolonged LOS and higher age-adjusted Charlson Comorbidity Index (CCI) associated with poorer outcomes [32]. The present study incorporates these established variables and further refines their predictive value by identifying critical thresholds—such as age-adjusted CCI greater than 7, LOS over 11 days, complications requiring additional surgery, and traumatic or thoracic procedures—that markedly increase readmission risk. The scoring system also identifies low-risk patients who might safely avoid routine testing, potentially reducing healthcare costs [[35], [36], [37], [38]]. Moreover, the detailed, clinically nuanced scoring system includes nonmodifiable factors, aiding proactive resource allocation for high-risk patients after discharge. Additionally, it could be used administratively to retroactively scale departmental readmission penalties, helping to avoid penalizing surgical teams for unavoidable readmissions among patients with excessively high-risk profiles lacking optimizable factors.

Machine learning has previously been applied to spine surgery for risk assessment. The Risk Assessment and Prediction Tool (RAPT), initially developed for hip and knee arthroplasty, utilized patient factors such as age, gender, mobility, and social supports to predict discharge destination [39]. RAPT was later adapted to predict length of stay (LOS) and 30-day readmission in lumbar and cervical spine surgery patients [40,41]. However, RAPT does not include key demographic, technical, or postoperative hospital course variables, likely due to its design for patient self-administration rather than detailed provider calculation.

Similarly, other scoring systems based on NSQIP (National Surgical Quality Improvement Project) data have been developed to predict spine surgery readmissions using preoperative risk factors, assigning points for each identified risk factor [42]. These systems, however, excluded significant variables such as prior year hospitalizations and postoperative LOS, which have been shown to impact readmission risk [16,34]. Furthermore, large multi-institutional databases, such as NSQIP, may have reduced accuracy and granularity compared to smaller, manually collected databases [43]. Another model from 2019 included demographic and preoperative comorbidities but lacked perioperative and postoperative factors essential for accurate readmission prediction [44]. In contrast, the current study’s risk calculator integrates relevant demographic, perioperative, and postoperative factors supported by recent literature, allowing for individualized patient risk assessment [11,18,34,45,46].

This study has several limitations. First, it was conducted at a single institution with a relatively small cohort, limiting the generalizability of the results. Variability in surgical techniques and perioperative protocols among the surgeons may have further influenced outcomes. Although known readmission risk factors such as age, sex, surgical indication, postoperative complications, and length of stay were controlled for, other potentially important variables like socioeconomic status, smoking status, medication adherence, and health literacy were unavailable and thus unaccounted for in the analysis. Additionally, the supervised-learning regression model used is reliant on high-quality data and susceptible to overfitting. The reported predictive performance (AUC) was calculated from the development dataset without independent external validation, potentially overestimating its predictive accuracy. Finally, patient-reported or functional outcomes, which may subjectively impact readmission risk, were not reliably available in our dataset and therefore were not evaluated. Future studies should include external validation and additional relevant factors to enhance the robustness and generalizability of the risk calculator.

## Declaration of competing interests

One or more of the authors declare financial or professional relationships on ICMJE-NASSJ disclosure forms.
